# Acceleration of ammonium phosphate hydrolysis using TiO_2_ microspheres as a catalyst for hydrogen production†

**DOI:** 10.1039/d0na00204f

**Published:** 2020-04-06

**Authors:** Ayman H. Zaki, Ahmed Esmail Shalan, Aya El-Shafeay, Yasser M. Gadelhak, Enas Ahmed, M. O. Abdel-Salam, M. Sobhi, S. I. El-dek

**Affiliations:** Materials Science and Nanotechnology Department, Faculty of Postgraduate Studies for Advanced Sciences (PSAS), Beni-Suef University Beni-Suef Egypt ayman.zaki@psas.bsu.edu.eg; Central Metallurgical Research and Development Institute P.O. Box 87 Helwan 11422 Cairo Egypt a.shalan133@gmail.com ahmed.shalan@bcmaterials.net; Renewable Energy Science and Engineering Department, Faculty of Postgraduate Studies for Advanced Sciences, Beni-Suef University Egypt; Egyptian Petroleum Research Institute P.O. 11727 Nasr City Cairo Egypt; BCMaterials-Basque Center for Materials, Applications and Nanostructures, Martina Casiano UPV/EHU Science Park, Barrio Sarriena s/n Leioa 48940 Spain

## Abstract

Titania microspheres are considered an adequate material with low cost and easily attainable pathways, and can be utilized in photocatalytic H_2_ production to solve the energy crisis. Spherical porous titanium dioxide materials, with nanostructure composition, were chemically synthesized from titanate nanotubes *via* a simple hydrothermal technique, then added as a catalyst to accelerate the route of ammonium phosphate hydrolysis for hydrogen production. The mechanism of sphere formation from titanate nanotubes is elucidated in detail through the current study. The prepared materials were applied as a photocatalyst to facilitate the separation and transfer of photoinduced electrons, while preventing the recombination of electron–hole pairs. Experimental results show that the obtained microspheres possess significantly enhanced photocatalytic hydrogen (H_2_) production performance. The amount of photocatalytic hydrogen product using the microspheres is found to be ∼2.5 fold greater than that of titanate nanotubes. Analytical techniques such as field-emission scanning electron microscopy (FESEM), high-resolution transmission electron microscopy (HR-TEM), simulated visible solar light and X-ray diffraction (XRD) were used for the evaluation and characterization of the developed products, as well as the elucidation of the route of hydrolysis in the hydrogen production process.

## Abstract 2

Titania microspheres are considered as an adequate material with low cost and easily attainable pathways, and can be utilized in photocatalytic H_2_ production to solve the energy crisis.

## Graphical abstract

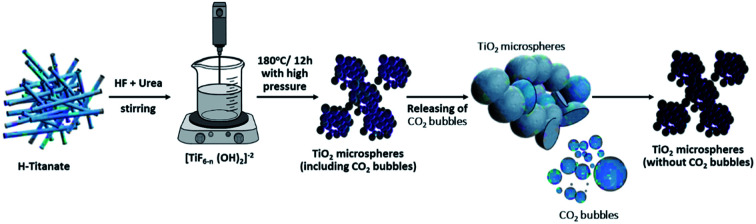

## Introduction

Hydrogen (H_2_) gas is one of the most significant lifesaving ways to solve the problem of fossil fuel depletion; it is produced by various technologies, such as steam reforming, partial oxidation and autothermal reforming.^1^ Most of the previous pathways to produce hydrogen are non-renewable, with products of low purity. Therefore, nowadays, different renewable technologies are used to produce hydrogen with high purity, such as water electrolysis^2,3^ and water photosplitting.^4,5^ The reasons limiting the wide use of hydrogen gas as fuel include its storage in tanks (liquid hydrogen or compressed gas), which is unsafe, and the high cost of its production. Consequently, recent research has focused on the storage of hydrogen in solid materials such as carbon nanotubes,^6^ graphite nanofibers,^7^ metal–organic frameworks^8^ and metal hydrides.^9–12^ On the one hand, ammonium phosphate has some advantages such as that it is cheap, stable, nontoxic and has a high hydrogen content, which make it a good source to release hydrogen and which have directed many research studies, including our current work, to focus on this area.^13,14^ It is produced by a reaction process between phosphoric acid (H_3_PO_4_) and anhydrous ammonia (NH_3_), which is very simple, low-cost pathway. The method of thermal extraction is not widely used because the total amount of hydrogen released is very small, so hydrolysis *via* catalysis is the most favorable way to extract hydrogen from ammonium phosphate.^13–15^ On the other hand, titanium dioxide (titania) is widely used as an effective photocatalyst due to its good photocatalytic and optical properties.^13,16^ Despite the wide band gap range of the different phases of titania, its efficiency is still limited because of the fast recombination and poor absorption of visible light (solar radiation contains 55.4% visible radiation). In addition, different morphological structures, such as nanoparticles,^17^ nanofibers,^18^ nanowires,^19^ nanotubes^20^ and nanospheres,^21^ can be easily attained for titanium dioxide. One of these promising structures are microspheres, which show amazing properties, through their high surface area, large pore size and pore volume.^22,23^ Titanium dioxide microsphere materials can be prepared by different methods, including hydrothermal, sacrificial templating, precipitation, sol–gel, gas bubbling and microemulsion.^24–29^ Further, it is noticed that the presence of pores in titanium dioxide spherical particles enables the increase in their reaction rate with other materials.^30^ Therefore, we used these spherical porous materials to accelerate the process of ammonium phosphate hydrolysis. Herein, we synthesized titanium dioxide microspheres using an easy hydrothermal process from titanate nanotubes as a precursor in the presence of hydrofluoric acid (HF) and urea, with some modifications compared to our previous study, to obtain more advanced materials suitable for different applications.^31^ Furthermore, the mechanism of microsphere formation was investigated in detail by changing the reaction constituents. Subsequently, we examines the efficiency of the obtained titania microspheres as a catalytic material towards the hydrolysis of tri-ammonium phosphate under visible light to release hydrogen. The obtained data confirmed that the synthesized TiO_2_ microsphere materials showed a promising performance as H_2_ production photocatalysts compared to the aggregate and fused spherical materials.

## Materials and methods

### Materials

Ammonium phosphate, titanium diisopropoxide, sodium hydroxide, ammonium hydroxide, absolute ethanol and urea were purchased from Sigma Aldrich, all were high purity reagents.

### Preparation of TiO_2_ microspheres

As in our recently published work with a slight modification,^31^ the obtained white milky solution, which was formed by adding 250 mL of 10 M NaOH to 5 g of anatase TiO_2_, was placed in a Teflon autoclave and heated at 160 °C for 23 h.^28,31^ After that, we added an amount of distilled water as well as 0.1 M HCl to the resultant white product of sodium titanate nanotubes (Na-TNTs) to attain H-titanate nanotubes (H-TNTs), which were washed with distilled water and finally dried at 80 °C for 2 h. Additionally, the prepared H-titanate nanotubes were calcined at 500 °C for 4 h in air. The calcined powders were mixed with 650 mL of distilled water using a magnetic stirrer for 30 min. Then, the same experiment was repeated several times with the addition of 10 mL HF as well as different amounts of urea (0, 5, 10 and 20 g), respectively. The samples of titania doped with urea are designated by the following abbreviations: u_0, u_5, u_10, and u_20 for 0, 5, 10 and 20 g of urea, respectively. In addition, urea was replaced with an equivalent amount of ammonium hydroxide to study its effect and to confirm the comparison between adding these two additives to the preparation solution. After continuous stirring for 1 h, a slightly turbid solution is obtained. This mixture was divided into two portions. The first portion was placed into a Teflon-lined stainless steel autoclave with 500 mL capacity to prepare the first sample. The second portion was filtered using filter paper, then was placed into another autoclave with the same capacity to prepare the other sample. The two autoclaves were kept at 180 °C for 12 h, then cooled to room temperature. The final steps of the hydrothermal route are filtration, washing the obtained powder with deionized water, and drying it at 80 °C for 2 h.

### Physical description of the obtained materials

The obtained samples were characterized and analyzed using different techniques, including field-emission scanning electron microscopy (FESEM, Quanta FEG 250, Switzerland) and high resolution transmission electron microscopy (HRTEM, JEOL-JEM 2100, Japan) to determine the surface morphologies and microstructures of the samples. In addition, the crystal structures of these samples were recorded using X-ray diffraction (XRD) with a PANalytical (Empyrean) X-ray diffractometer with Cu Kα1 radiation (wavelength 1.5406 Å) at an accelerating voltage of 40 kV, current of 30 mA, scan angle range of 5–80° and scan step of 0.02°. Additionally, ImageJ as well as Normalizing Histogram Origin Programs were used to calculate the particle size distributions of the obtained nanoparticles.

### Photocatalytic performance experiments through ammonium phosphate hydrolysis

The efficiency of the photocatalyst was estimated by measuring the volume of hydrogen gas released from tri-ammonium phosphate hydrolysis. All of the experiments were carried out under solar light using a magnetic stirrer at a speed of 700 rpm at ambient temperature. A fixed weight ratio of tri-ammonium phosphate to titania photocatalyst (2 : 1) was added to distilled water. In brief, 0.1 g of tri-ammonium phosphate was well dissolved in 100 mL of distilled water, placed in a reaction flask, then 0.05 g of titania photocatalyst was added to the solution and connected to a cylinder filled with water to measure the volume of hydrogen gas released from the reaction. The volume of hydrogen gas evolved was checked by measuring the displacement of water level every minute. The experiment was stopped when no more hydrogen gas release was observed. In addition, to confirm the effect of the titanate materials as a catalyst for the hydrolysis reaction, we constructed the same experiment but without any catalytic material, as a control experiment, and repeated the reaction to observe that no appreciable amount of hydrogen gas evolved.

## Results and discussion

**Fig. 1:**
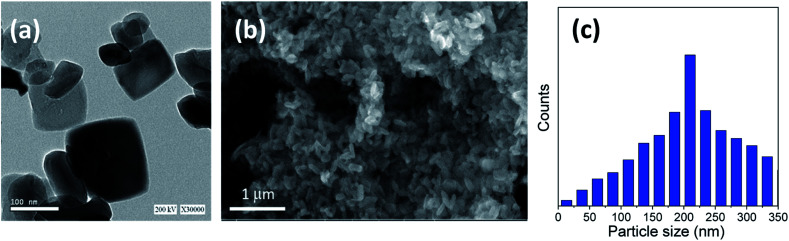
(a) HRTEM, (b) FESEM image and (c) particle size distribution of the prepared sample (u_0) without the urea additive.

The titania microspheres were prepared from titanate nanotubes, and the addition of urea as well as ammonia for surface and particle size modification was also studied. Firstly, the morphology and particle size distribution for the pure titania microspheres, without urea additive (u_0), were checked *via* HRTEM and FESEM, as shown in Fig. 1a–c. Fig. 1a shows the HRTEM of the obtained powders, confirming the formation of the particles in nanoscale range. In addition, Fig. 1b shows the homogenous aggregation of the obtained powders with particle size range of ∼150–300 nm, and most of the obtained particles were ∼200 nm, as confirmed from the particle size distribution measurements shown in Fig. 1c.

Furthermore, different structure parameters for the obtained titania microspheres from titania nanotubes were checked and confirmed *via* different calculations, as mentioned in our recent study on the same materials.^31^ The density was found in the range between 3.8709–3.8946 g cm^−3^; the calculated crystalline size was in the range of 49.3–63.7 nm; the pore volume was in the range of 0.0404–0.0445 cm^3^ g^−1^; the surface area was in the range of 20.2–27.5 m^2^ g^−1^ and the band gap calculated to be 3.25 eV.^31^

**Fig. 2:**
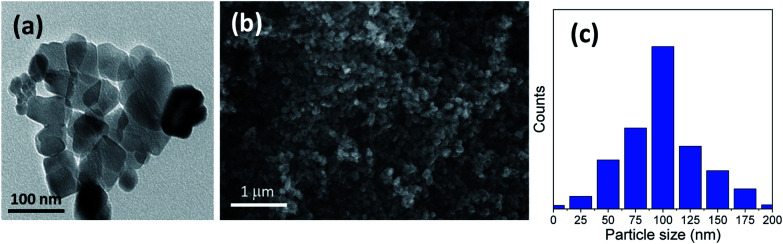
Morphology characterization through (a) HRTEM and (b) FESEM micrograph image; (c) particle size distribution of the prepared microspheres sample using 5 g (u_5) of urea additive.

**Fig. 3:**
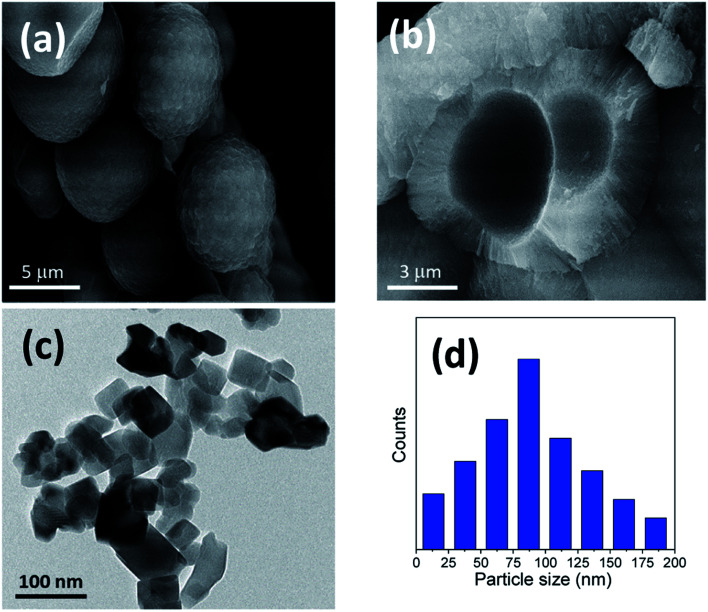
Morphology of the prepared samples: (a) FESEM micrograph of the formed microspheres with 10 gm of urea (u_10); (b) close-up on some broken spheres; (c) HRTEM micrograph; (d) particle size distribution of the same sample (u_10).

Furthermore, Fig. 2 and 3 show the obtained HRTEM images as well as FESEM images for samples with the addition of 5 and 10 g of urea (u_5 & u_10), respectively. Fig. 2a and b shows the formation of TiO_2_ microspheres for the sample with 5 g of urea (u_5), while Fig. 3a and b shows the formation of TiO_2_ microspheres for the 10 g of urea (u_10) sample, with the calculated particle size distribution for both samples, as illustrated in Fig. 2c and 3d.

The addition of urea results in enhancement in the morphologies and structures of both the u_5 and u_10 samples. From the obtained data, and by calculating the particle size distributions of both samples, we noted that the particle size of the spherical materials obtained by adding urea as additive is lower than that of the pure sample without adding urea. Moreover, it is clear from the obtained results that urea is essential for the formation of the desired microspheres, and removing or exchanging urea with ammonia as a source of hydroxyl groups has no benefit in this case.

In the sample with 10 g urea (u_10), the spheres have smooth surface and diameters ranging from ∼2.8 to 5.3 μm. Fig. 3b shows a close-up on two broken spheres. It can be observed that the formed spheres have a solid wall and a hollow interior. The incompletely formed spheres have diameters ranging ∼2.1 to 3.7 μm and possess a smooth surface. Every large sphere contains several small particles that can detected and confirmed from the obtained HRTEM images, with a particle size range of ∼80–100 nm in the case of u_5 samples, and ∼70–80 nm in the case of u_10 samples, as confirmed from the particle size distribution measurements shown in Fig. 2c and 3d.

**Fig. 4:**
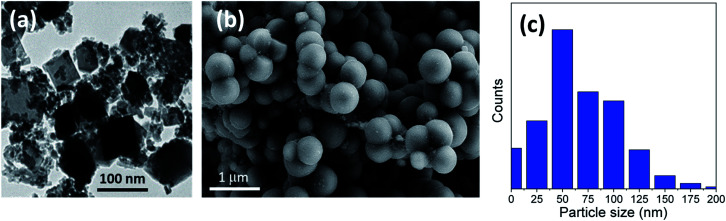
(a) HRTEM, (b) FESEM image and (c) particle size distribution of the prepared sample using 20 gm of urea (u_20) as an additive.

The ideal case to prepare titania microspheres with the optimized particle size was detected in the addition of 20 gm of urea to the solution during the reaction process. Fig. 4a shows the HRTEM micrograph for the nanosheets comprising the sphere walls. It is evident that there exists a wide size distribution of lateral dimensions of the formed sheets. The large-sized sheets have dimensions of ∼113–118 nm, while the medium sheets have dimensions ranging from ∼62–81 nm. In addition, the sizes of the small sheets range from ∼32–52 nm, with even smaller clusters present, as confirmed in Fig. 4c. In addition, Fig. 4b shows that the walls of the hollow spheres are formed of aggregated TiO_2_ nanosheets that have been self-assembled to form the walls of the microspheres. As shown, the microspheres are not only individually self-assembled, but are also rather fused into neighboring spheres.

We can conclude that using lower quantities of urea did not yield any spheres, but only TiO_2_ aggregates, as shown in Fig. 1 for the bare sample without urea (u_0), as well as in Fig. 2 and 3 for u_5 and u_10 samples, respectively. The lack of formation of any microspheres shows the crucial role of the presence of urea for the self-assembly of the nanosheets. These results also show the critical role of urea concentration on the formed microsphere; there exists a critical concentration below which no spheres will be formed.

The following section provides deeper insights to further understand the role of urea in the self-assembly of the nanosheets in the form of microspheres.

When titania nanotubes (TNTs) are mixed with HF, the tubes will be eroded by the fluoride ions forming TiF_6_^2−^ ions, as represented by eqn (1).^32^

1 TNTs + HF → TiF_6_^2−^ + H^+^ + H_2_O

When subjected to the hydrothermal conditions, urea thermally decomposes, yielding hydroxyl ions, as shown in eqn (2).^33^

2 (NH_2_)_2_CO + H_2_O → CO_2_ + NH_4_^+^ + OH^−^

**Fig. 5:**
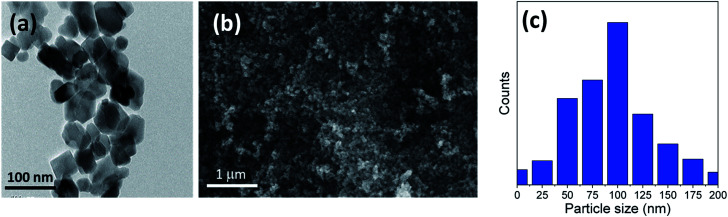
(a) HRTEM, (b) FESEM image and (c) particle size distribution of the prepared sample using ammonia instead of urea as an additive.

The generated hydroxyl ions will neutralize the formed protons, thereby affecting the pH of the solution and, consequently, the rate of hydrolysis of the TiF_6_^2−^ ions. Previous studies confirm the effect of urea dosage on the formation of TiO_2_ microspheres and claimed that the presence of large quantities of urea will produce larger quantities of hydroxyl ions, thereby increasing the hydrolysis rate, which yields microspheres.^32^ However, for lower quantities of urea, the hydrolysis rate will become slow, and only aggregates will be formed, but no spheres. This dual role of urea (change pH and rate of hydrolysis) has been supported by several previous studies.^33–35^ For the current study, urea has been replaced by an equivalent quantity of ammonium hydroxide (ammonia), but no spheres were formed in the final product. As shown in Fig. 5, only small aggregates could be observed, with no evidence of the formation of self-assembled microspheres. This shows that the role of urea is not limited to being an *in situ* hydroxyl ion producer, but rather, the produced carbon dioxide also has a direct contribution to the formation of microspheres. It is believed that the formed CO_2_ bubbles evolving in the solution can act as a “soft template” for the assembly of the nanosheets.

Subsequently, different images have been checked for the samples with and without urea, after replacing urea with ammonia, with different magnifications and checking the positions for every sample of u_0, u_20 and ammonia additive, for the samples shown in Fig. S1, S2 and S3 (in the ESI†), respectively.

**Scheme 1:**
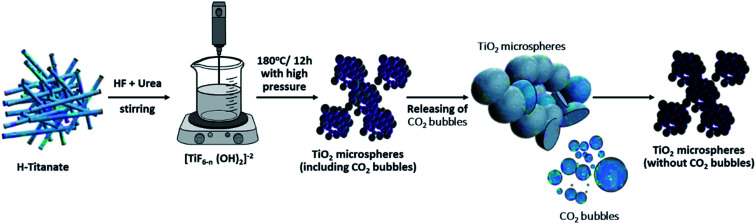
Graphical representation for the mechanism of formation of TiO_2_ microspheres from titanate nanotube microstructures.

Several studies have reported a similar explanation for the formation of microspheres when using urea in the hydrothermal precursor.^33–35^ For instance, Zhou *et al.* prepared hollow Sn_2_Nb_2_O_7_ microspheres using urea as a source of bubbles, which will act as a template for the formation of the microspheres.^36^ Similarly, urea was used as a source of bubbles to solvothermally produce microspheres of numerous materials, such as BiVO_4_,^37^ K_4_Nb_6_O_17_,^38^ hydroxyapatite,^39,40^ ZnO,^41^ Fe_3_O_4_,^42^ NiCo_2_S_4_,^43^ Ca_2_Ge_7_O_16_ (ref. 44) and NiO.^45^ All such reported studies supported the “soft template” mechanism of the produced CO_2_ bubbles for the self-assembly of the produced aggregates. For the current study, it is believed that the quantity of urea and thereby that of the CO_2_ bubbles will directly affect the morphology of the product TiO_2_. When the quantity of urea is still below a critical value, the quantity of produced bubbles is low and not sufficient to form self-assembled microspheres. At certain urea concentrations, the nanosheets will start assembling in the form of microspheres over the surface of the CO_2_ bubbles formed. It is believed that the formed microspheres will colloid with each other and continue growing while fused to each other (*i.e.*, incomplete spheres attached to each other). When the quantity of urea is further increased, it is assumed that the extra CO_2_ bubbles will have a shearing effect on the microspheres, thereby breaking any fusion and allowing the spheres to grow individually and independently of the neighboring spheres. The self-assembled nanosheets are thought to undergo a dissolution–recrystallization mechanism, whereby the fluoride ions in solution chemically etch the core of the formed spheres.^35^ The dissolved ions are thought to be recrystallized at the surface of the spheres. The mechanism of formation is graphically represented in Scheme 1.

**Fig. 6:**
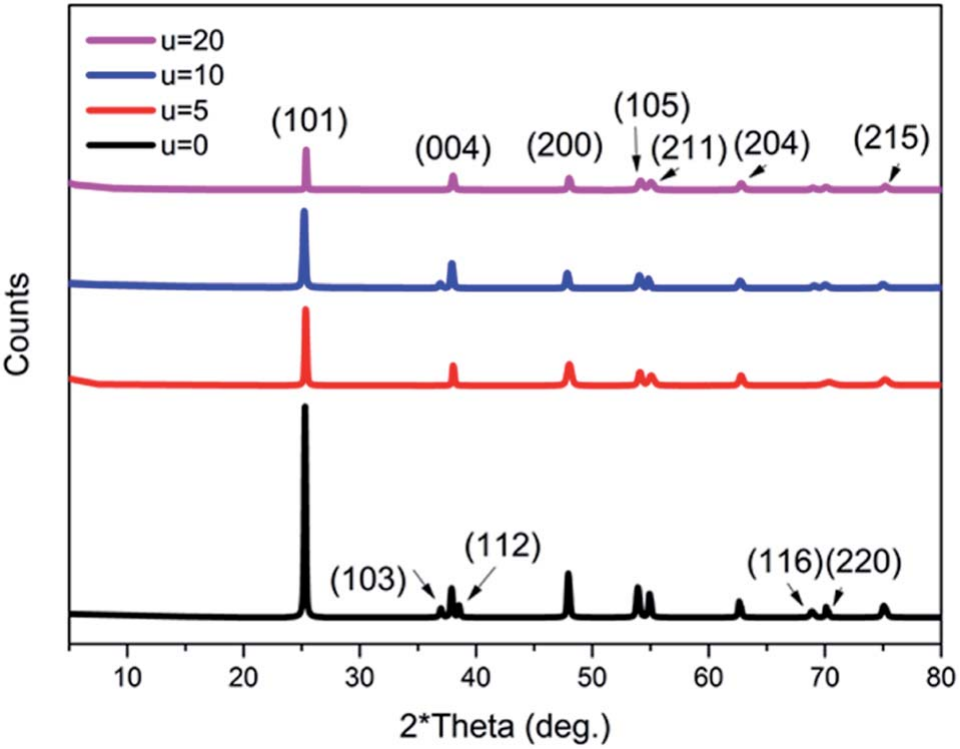
XRD patterns of all of the prepared titania microstructure samples in the presence of different urea concentrations (u_0, u_5, u_10, u_20).

Moreover, it is believed that the nanosheets undergo an Ostwald ripening process, whereby smaller sheets dissolve and start forming larger ones.^46^ It is assumed that this process is responsible for the wide size distribution of the nanosheets observed. Ostwald ripening is also assumed to be the significant mechanism that aids the hollowing of the microspheres.^15,47^ The XRD patterns of all of the prepared samples with the addition of different concentrations of urea (u_0, u_5, u_10, u_20) are shown in Fig. 6. All of the samples show a pure anatase phase, which corresponds to ICDD card number 01-075-2545.

**Fig. 7:**
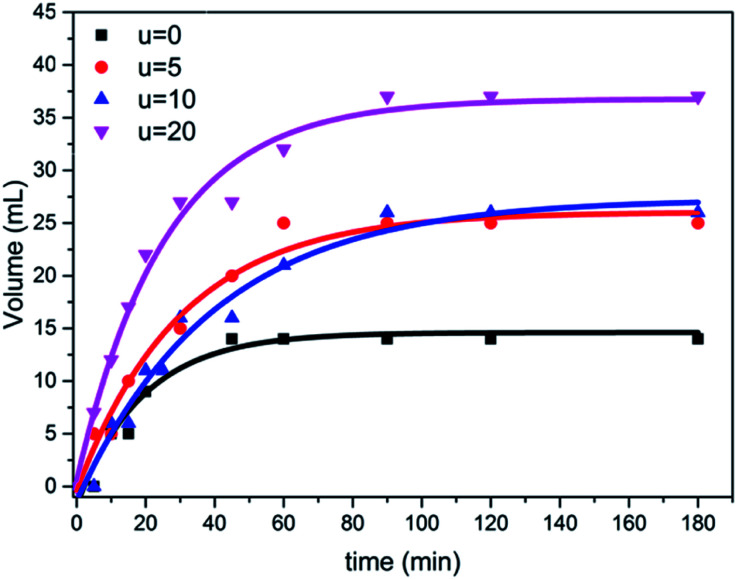
Photocatalytic activity through ammonium phosphate hydrolysis with regard to hydrogen release from all the prepared titania microstructure samples in the presence of different urea concentrations (u_0, u_5, u_10, u_20).

The photocatalytic activity of the prepared samples with different amounts of urea were tested for the hydrolysis of ammonium phosphate with regard to hydrogen evolution process under simulated visible solar light, using a mercury lamp with an intensity of 1000 W. Furthermore, the obtained results from the hydrolysis reaction shows how the hydrogen gas was released during the reaction, confirming the good separation and transfer of photoinduced electrons, thus decreasing the rate of recombination of electron–hole pairs. As shown in Fig. 7, the sample (u_20) yielded the highest volume of hydrogen gas, which is approximately 2.5 folds higher than that of the TiO_2_ aggregates formed without urea addition; *i.e.*, in the case of titania nanotube samples. This can be attributed to the higher light-harvesting ability of self-assembled microspheres due to the multiple reflections of incident light and improved photon absorption^48,49^ compared to individual aggregates. The individual microspheres in the u_20 sample also show an improved performance compared to the fused spheres in the u_10 sample, which could be attributed to the higher surface area-to-volume ratio and shorter mass transport lengths of the individual microspheres.^50,51^

## Conclusion

In this study, a simple hydrothermal pathway was utilized to synthesize anatase TiO_2_ microspheres *via* titanate nanotubes as the starting material, in the presence and absence of urea and ammonia as additive materials. Furthermore, the prepared titania materials under different conditions with and without urea, as well as ammonia, were characterized and studied using several techniques. As well, the results show that the solution pH can be affected and changed by adding urea during the preparation steps. The efficiently prepared titania microstructure materials, with catalytic behavior, work as photocatalysts to enhance the hydrolysis of tri-ammonium phosphate under visible light, with the benefit of accelerating the reaction process in order to facilitate the production of hydrogen (H_2_) during the reaction. Additionally, the CO_2_ bubbles produced during the hydrolysis reaction is believed to play an important role in the self-assembly of the TiO_2_ microspheres. Moreover, the mechanism of microsphere formation was discussed in detail based on changing the reaction constituents. We can conclude from the obtained data that the synthesized TiO_2_ microsphere materials showed promising performance as H_2_ production photocatalyst compared to the aggregates and the fused spherical materials.

## Conflicts of interest

The authors declare no conflict of interest.

## Supplementary Material

NA-002-D0NA00204F-s001
