# Expression of concern: Acceleration of ammonium phosphate hydrolysis using TiO_2_ microspheres as a catalyst for hydrogen production

**DOI:** 10.1039/d4na90040e

**Published:** 2024-03-22

**Authors:** Ayman H. Zaki, Ahmed Esmail Shalan, Aya El-Shafeay, Yasser M. Gadelhak, Enas Ahmed, M. O. Abdel-Salam, M. Sobhi, S. I. El-dek

**Affiliations:** a Materials Science and Nanotechnology Department, Faculty of Postgraduate Studies for Advanced Sciences (PSAS), Beni-Suef University Beni-Suef Egypt ayman.zaki@psas.bsu.edu.eg; b Central Metallurgical Research and Development Institute P.O. Box 87, Helwan 11422 Cairo Egypt a.shalan133@gmail.com ahmed.shalan@bcmaterials.net; c Renewable Energy Science and Engineering Department, Faculty of Postgraduate Studies for Advanced Sciences, Beni-Suef University Egypt; d Egyptian Petroleum Research Institute P.O. 11727, Nasr City Cairo Egypt; e BCMaterials-Basque Center for Materials, Applications and Nanostructures Martina Casiano, UPV/EHU Science Park, Barrio Sarriena s/n Leioa 48940 Spain

## Abstract

Expression of concern for ‘Acceleration of ammonium phosphate hydrolysis using TiO_2_ microspheres as a catalyst for hydrogen production’ by Ayman H. Zaki *et al.*, *Nanoscale Adv.*, 2020, **2**, 2080–2086, https://doi.org/10.1039/D0NA00204F.

The Royal Society of Chemistry is publishing this expression of concern in order to alert readers that concerns have been raised regarding the reliability of the particle size distribution data in Fig. 1, and the SEM images in Fig. 2b and 5b. An investigation is underway, and an expression of concern will continue to be associated with the article until a final outcome is reached.

Jeremy Allen

19th March 2024

Executive Editor, *Nanoscale Advances*

## Supplementary Material

