# Supplementary material for: Acceleration of ammonium phosphate hydrolysis using TiO2 microspheres as a catalyst for hydrogen production
Source: Nanoscale Adv. 2020 Apr 6;2(5):2080–6. doi: 10.1039/d0na00204f (PMC9416965; doi:10.1039/d0na00204f)
Supplement: NA-002-D0NA00204F-s001 [file NA-002-D0NA00204F-s001.pdf]

Electronic Supplementary Material (ESI) for Nanoscale Advances.
This journal is © The Royal Society of Chemistry 2020

Acceleration of Ammonium Phosphate Hydrolysis Process via TiO_2 Microspheres as A Catalyst for Hydrogen Production

Ayman H. Zaki[a†], Ahmed Esmail Shalan[b,e*†], Aya El-Shafeay[a], Yasser M. Gadelhak[a], Enas Ahmed[c], M. O. Abdel-Salam[d], M. Sobhi[b], and S. I. El-dek[a]*

[a] Materials Science and Nanotechnology Department, Faculty of Postgraduate Studies for Advanced Sciences (PSAS), Beni-Suef University, Beni-Suef, Egypt

[b] Central Metallurgical Research and Development Institute, P.O. Box 87, Helwan, 11422, Cairo, Egypt.

[c] Renewable Energy Science and Engineering Department, Faculty of Postgraduate Studies for Advanced Sciences, Beni-Suef University, Egypt

[d] Egyptian Petroleum Research Institute, Nasr City, P.O. 11727, Cairo, Egypt

[e] BCMaterials-Basque Center for Materials, Applications and Nanostructures, Martina Casiano, UPV/EHU Science Park, Barrio Sarriena s/n, Leioa 48940, Spain.

*Corresponding author: (A. H. Z.) E-mail: *ayman.zaki@psas.bsu.edu.eg;*

(A. E. S.) E-mail: *a.shalan133@gmail.com*; *ahmed.shalan@bcmaterials.net*

† The mentioned authors contributed equally to this manuscript.

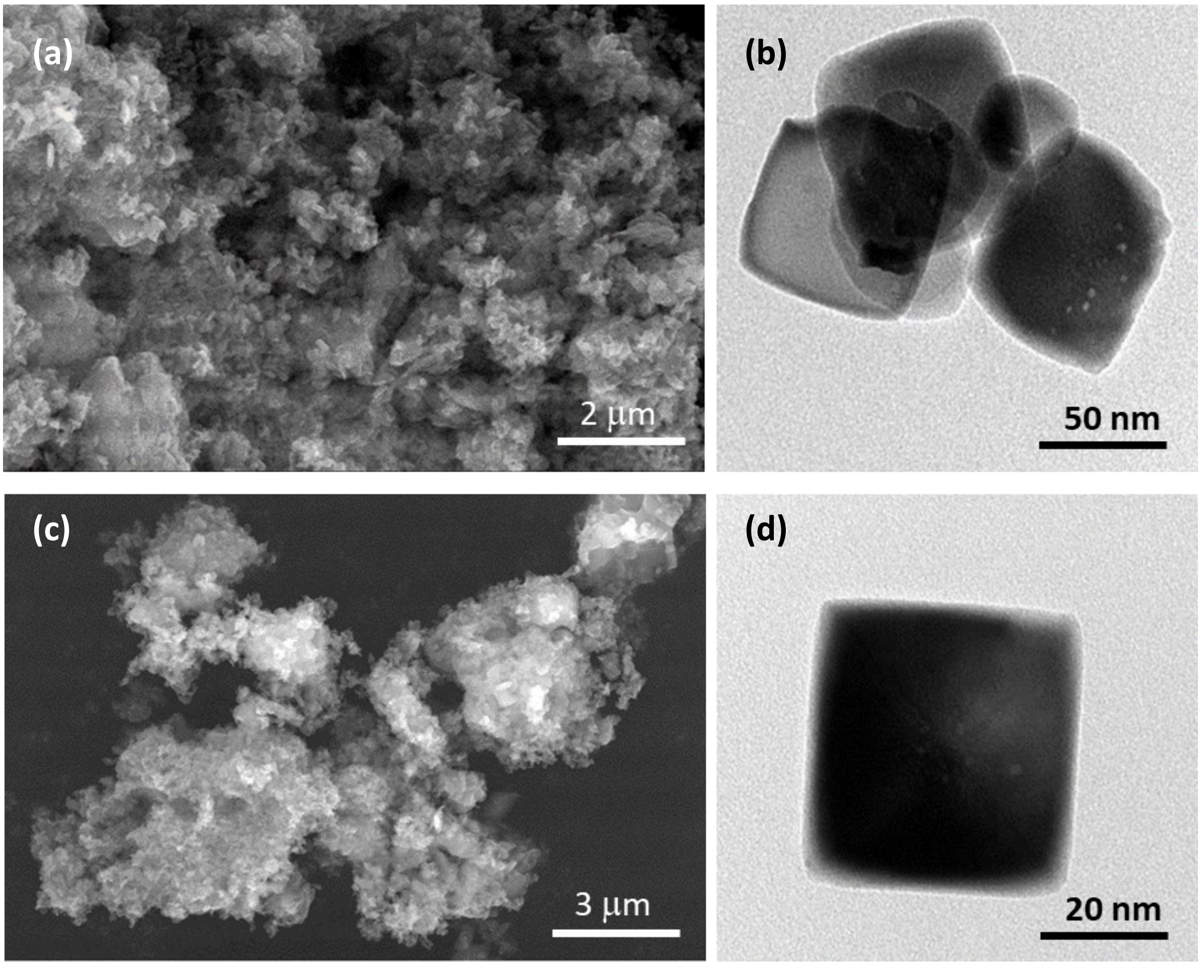

Figure S1: (a & c) FESEM and (b & d) HRTEM images of the prepared sample without of Urea additive (u_0) with different magnifications and positions.

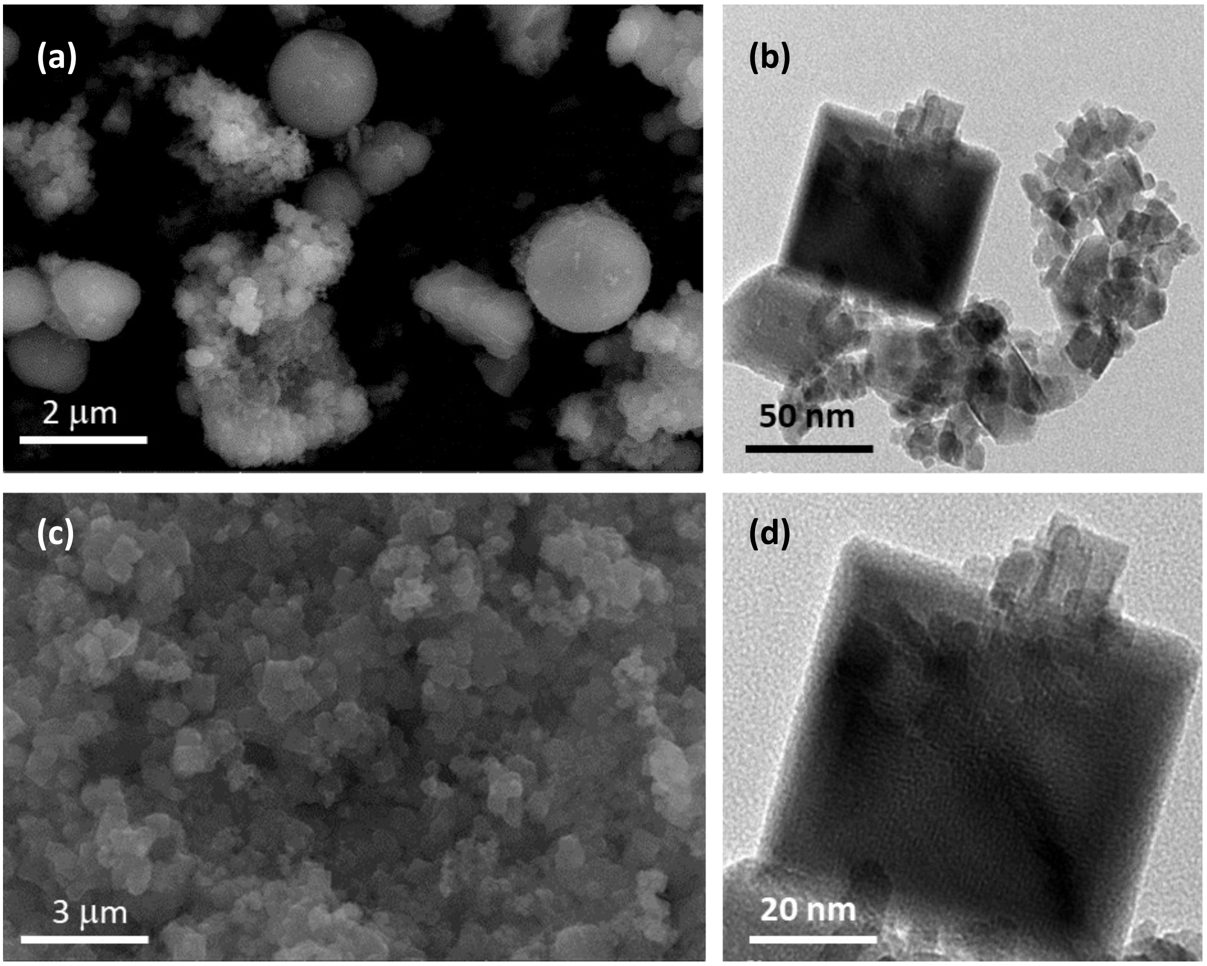
(a)
2 μm
(b)
50 nm
(c)
3 μm
(d)
20 nm

Figure S2: (a & c) FESEM and (b & d) HRTEM images of the prepared sample using 20 gm of Urea (u_20) with different magnifications and positions.

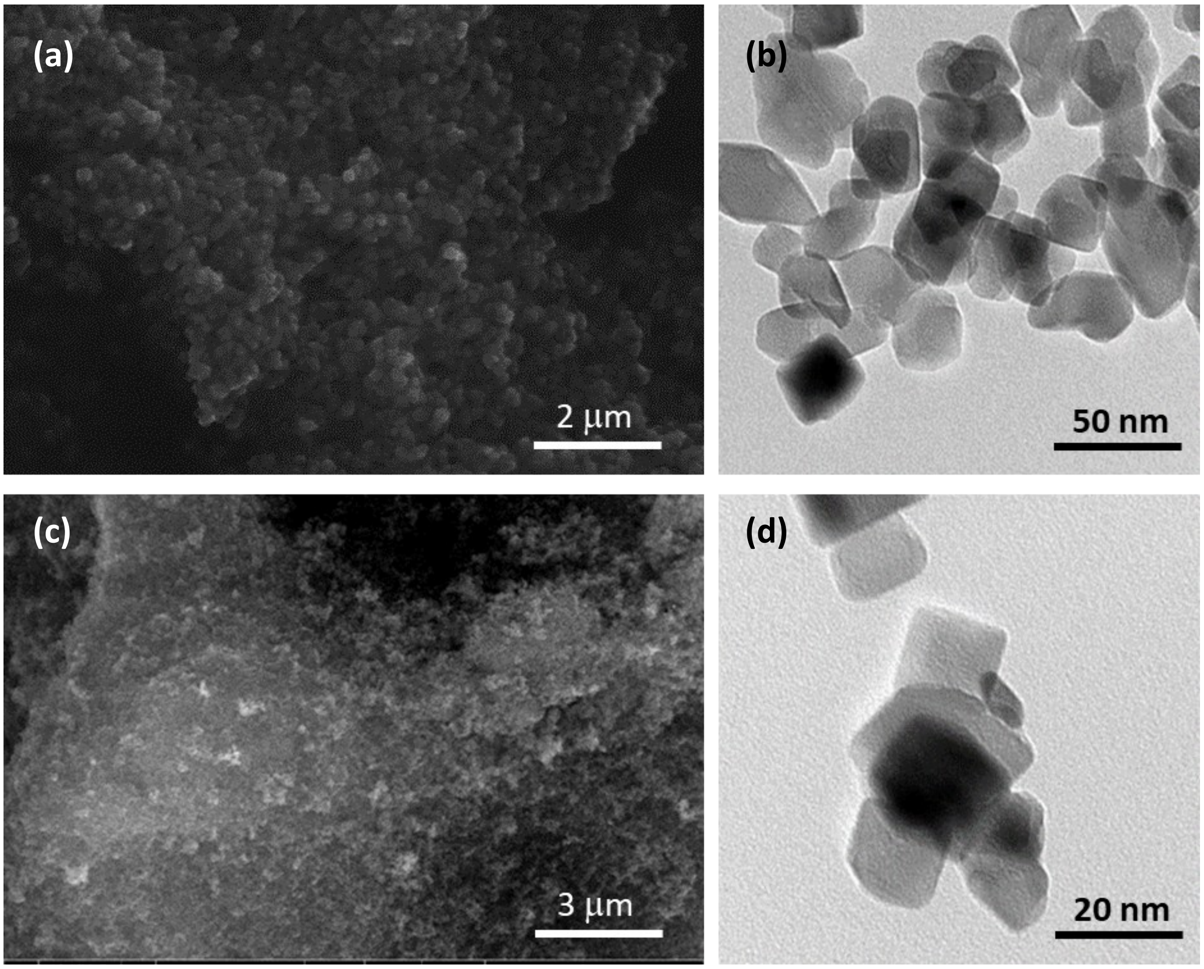

Figure S3: (a & c) FESEM and (b & d) HRTEM images of the prepared sample using ammonia instead of Urea additive with different magnifications and positions.
